# Quality of benzathine penicillin G: A multinational cross‐sectional study

**DOI:** 10.1002/prp2.668

**Published:** 2020-10-22

**Authors:** Robert M. Hand, S. M. D. K. Ganga Senarathna, Madhu Page‐Sharp, Katherine Gray, Dianne Sika‐Paotonu, Meru Sheel, Victor T. G. Chuang, Jorge Martinez, Giuseppe Luna, Laurens Manning, Rosemary Wyber, Jonathan R. Carapetis, Kevin T. Batty

**Affiliations:** ^1^ Wesfarmers Centre of Vaccines and Infectious Diseases Telethon Kids Institute University of Western Australia Perth Western Australia Australia; ^2^ School of Pharmacy and Biomedical Sciences Curtin University Bentley Western Australia Australia; ^3^ Dean’s Department and Department of Pathology & Molecular Medicine Wellington School of Medicine and Health Sciences University of Otago Dunedin New Zealand; ^4^ Faculty of Health Victoria University of Wellington Wellington New Zealand; ^5^ Maurice Wilkins Centre for Molecular Biodiscovery University of Auckland Auckland New Zealand; ^6^ School of Pharmacy Monash University Malaysia Selangor Malaysia; ^7^ Faculty of Health and Medical Sciences University of Western Australia Perth Western Australia Australia; ^8^ The George Institute for Global Health Sydney New South Wales Australia; ^9^ Department of Infectious Diseases Perth Children’s Hospital Perth Western Australia Australia

**Keywords:** benzathine benzylpenicillin, benzathine penicillin G, particle size analysis, pharmaceutical quality

## Abstract

Benzathine penicillin G (BPG) is used as first‐line treatment for most forms of syphilis and as secondary prophylaxis against rheumatic heart disease (RHD). Perceptions that poor quality of BPG is linked to reported adverse effects and therapeutic failure may impact syphilis and RHD control programs. Clinical networks and web‐based advertising were used to obtain vials of BPG from a wide range of countries. The quality of BPG was assessed using a high performance liquid chromatography assay capable of detecting relevant impurities and degradation products. Tests for water content, presence of heavy metals and physical characteristics of BPG, including particle size analysis and optical microscopy, also were conducted. Thirty‐five batches of BPG were sourced from 16 countries across 4 WHO regions. All batches passed the US Pharmacopeia requirements for BPG injection (content), with no evidence of breakdown products or other detected contaminants. Water content and heavy metal analysis (n = 11) indicated adherence to regulatory standards and Good Manufacturing Practice. Particle size analysis (n = 20) found two batches with aggregated particles (>400 µm) that were dispersed following sonication. Current batches of BPG were of satisfactory pharmaceutical quality but aggregated particles were found in a modest proportion of samples. Future studies should focus on the physical characteristics of BPG which may contribute to variations in plasma penicillin concentrations an observed needle blockages in clinical practice. Pharmacopeial monographs could be revised to include standards on particle size and crystal morphology of BPG.

## Graphical abstract

All 35 batches were of adequate pharmaceutical potency required by the United States pharmacopeial standards. Differences in crystal size between brands may explain variability in clinician experience, providing opportunities to improve manufacturing standards.

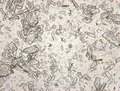

**Abbreviations**
BPG: Benzathine penicillin G
RHD: rheumatic heart disease
API: active pharmaceutical ingredient
GMP: Good Manufacturing Process

**What is already known about this subject**

- Benzathine penicillin G (BPG) has been used for decades to treat syphilis and for secondary prophylaxis against rheumatic fever.
- Concerns over product quality and adverse reactions have impacted clinician confidence.
- Limited research exists to explain observed pharmacokinetic differences between brands of BPG.

**What this study adds**

- This study analyzed 35 batches of commercially available BPG from 16 countries across four of the six WHO regions.
- Tested samples adhered to United States pharmacopeial standards without detectable contamination, providing reassurance to clinicians.
- Significant differences in particle size between brands may explain observed pharmacokinetic variability.

## 1 BACKGROUND

Benzathine penicillin G (BPG) has been on the World Health Organization (WHO) essential medications list since 1977 and is currently recommended as first‐line treatment of syphilis and for secondary prophylaxis for recurrent rheumatic fever.^1^, ^2^, ^3^, ^4^ Following intramuscular injection of BPG, the crystalline combination of penicillin G and benzathine (in a 2:1 molar ratio) is slowly absorbed and results in prolonged plasma concentrations of penicillin G.^5^, ^6^ Depending on the indication, BPG may be administered as weekly or monthly injections.^7^

Severe adverse reactions following BPG are rare, but occasionally fatal. Usually this occurs at the “end of the needle” and is assumed to be anaphylaxis.^8^ These reactions occur almost exclusively in the setting of significant rheumatic heart disease (RHD), rather than syphilis, suggesting a disease‐related mechanism or other patient factors, rather than a reaction to the BPG components.^8^, ^9^ However, anecdotal reports of poor quality BPG have been linked to severe adverse effects and needle blockages.^7^, ^10^ Any perceptions that BPG is a low‐quality medication are likely to impact on national syphilis and RHD control programs.^10^, ^11^

The production pathway of BPG is complex, with the active pharmaceutical ingredient (API) produced by primary manufacturers, and subsequently on‐sold to secondary pharmaceutical manufacturers for packaging, distribution, and sale.^12^ It is presumed that a complicated supply chain and low profit margins have led to a reduction of API manufacturers, although the exact number is unknown.^12^ Reduced commercial incentive for pharmaceutical companies to produce BPG has also led to delivery delays and stock‐outs.^13^ Of greater concern is possible breaches of Good Manufacturing Process (GMP) by some manufacturers, resulting in stock recalls and scrutiny of the manufacturing process.^12^ These factors have left RHD and syphilis control programmes with insecure supply and occasionally, prolonged BPG shortages.^7^ Supply challenges are not limited to lyophilized BPG, with stock‐outs occurring in countries which primarily utilize Bicillin L‐A^®^, a ready‐to‐use BPG injectable suspension. The widespread adoption of a ready‐to‐use preparation is constrained by high cost and reliance on a functional cold chain.^14^

Despite reports of needle blockages, few publications report the quality and physical characteristics of BPG.^7^, ^15^ A clinical study undertaken in the 1990s compared two brands of BPG in Egypt (one local and one imported) and reported significant pharmacokinetic differences between the formulations, using a microbiological assay (plate diffusion method). There were also anecdotal physical differences, with the locally‐produced preparation “more difficult to pass through an 18G needle”.^15^ More recently, a laboratory study showed markedly different crystal shapes between two brands of BPG, indicating that ‘needle‐like’ crystals were more likely to cause needle blockage than ‘plate‐like’ crystals, despite having equivalent crystal volume.^16^ The authors suggested reviewing the manufacturing process, to minimize the differences in crystal structure. However, there are no subsequent reports or comparative studies evaluating the physical characteristics, pharmacokinetics, or bioequivalence of BPG injectable formulations.

Against this background, and with a primary aim of determining whether clinicians in resource‐poor settings can have confidence in currently‐available supplies of BPG, we sought to investigate the pharmaceutical quality of powdered BPG injection sourced from a range of international clinical settings. We also conducted a thermal stability assessment of Bicillin L‐A^®^, due to anecdotal reports of this product being discarded after short periods outside refrigeration.

## 2 MATERIALS AND METHODS

### 2.1 Sample collection and testing rationale

Samples of powdered BPG injection were obtained through the investigators’ networks and the website of Reach, an international RHD technical support and advocacy organization, between 2015 and 2018. Participants were asked to supply a minimum of five, ideally 10, in‐date vials of BPG from a single batch available in their country. Smaller quantities were accepted if a shortage was present. Where possible, participants were asked to provide additional vials from other batches and multiple brands from the same country. As the intent was to sample BPG available at the community or end‐user level, there was no engagement with BPG manufacturers or distributors. Vials were shipped to Australia via courier or personal (aeroplane) luggage with the appropriate customs declarations and import licence, and stored < 25°C and protected from light.

All samples underwent testing for BPG content and the presence of breakdown products. A subset of samples selected at random were subjected to additional testing for pharmaceutical quality, including water content, melting point and heavy metal analysis (indicators of good manufacturing practice), and physical characteristics (light microscopy and particle size analysis).

### 2.2 Benzathine penicillin G analysis

A reversed‐phase, ion‐pairing high performance liquid chromatography (HPLC) method^17^ with minor modifications was adapted for the present study (see Supplementary information and Figures S1‐S3 for full details).

#### 2.2.1 Powder vials

Two aliquots of approximately 2 mg of powder (accurately weighed) from each BPG vial were dissolved in 1 mL dimethylformamide (DMF). Test solutions were prepared by diluting the stock solution in de‐ionized water to a nominal concentration of 0.2 mg/mL for analysis.

For batches of five BPG vials (n = 17), three vials were assayed and two were retained for other pharmaceutical tests. For batches of ten vials (n = 12), six vials were assayed (one batch of which was analyzed three months after the manufacturer's expiry date; all other vials were tested before the expiry date). Six vials were provided as individual samples from different batches or countries; each vial was assayed in triplicate (Table 1; source country and manufacturer not disclosed).

**Table 1:** Benzathine penicillin G (BPG) content, melting point, and particle size analysis of commercial BPG injection powder sourced from international clinical settings

Code	Number of Vials Tested	BPG content (%)^a^ (Mean ± SD)	Melting Point^b^ (°C)	Specific surface area (m^2^/g)	Average particle size; D50^c^ (µm)	D10‐D90^c^ (µm)
1	3	106.1 ± 0.7	126	0.94	15	3‐48
2	6	98.4 ± 2.5	124	0.55	33	5‐86
3	3	97.5 ± 0.4	124	0.48	23	6‐71
4	1	105.6	129	0.45	25	6‐90
5	3	106.4 ± 3.3	125	0.39	32	8‐89
6	3	105.8 ± 1.8	131	0.51	22	6‐62
7	6	107.0 ± 1.4	129	0.52	37	5‐95
8	6	106.6 ± 2.7	129	0.54	22	6‐63
9	6	106.3 ± 2.6	133	0.45	27	7‐71
10	3	99.0 ± 1.3	124	1.33	8	2‐34
11	6	97.2 ± 1.6	123	0.37	33	11‐91
12	3	104.6 ± 2.4	127	0.39	30	8‐90
13	1	105.3	128	0.37	33	8‐99
14	3	106.9 ± 2.1	128	0.55	20	6‐55
15	3	106.8 ± 1.8	126	0.94	18	3‐50
16	3	106.1 ± 0.8	132	1.07	18	2‐59
17	3	103.4 ± 2.5	132	0.92	11	3‐34
18	3	98.3 ± 1.1	124	1.11	10	3‐34
19	3	98.1 ± 0.4	124	1.03	11	3‐35
20	6	98.0 ± 1.0	126	1.01	11	3‐36
21	6	91.5 ± 1.1	127			
22	6	97.3 ± 1.2	126			
23	3	100.2 ± 0.4	126			
24	3	101.9 ± 2.4	128			
25	3	101.9 ± 2.4	127			
26^d^	3	98.5 ± 1.0	127			
27^d^	3	99.9 ± 3.4	128			
28	6	102.7 ± 5.0	127			
29	1	100.8	128			
30	6	108.1 ± 7.4	126			
31	6	98.5 ± 0.9	124			
32	1	102.6	127			
33	1	101.4	129			
34	1	101.6	128			
35^e^	6	95.8 ± 5.1^e^	126			

^a^ Pharmacopeial requirements are 90%‐115% BPG content for the injectable suspension in the United States Pharmacopeia and 94.5%‐102% for the active pharmaceutical ingredient (API) in the British Pharmacopoeia. The BPG content in the powder for injection is expressed as percent of the nominal amount, based on the penicillin G concentration and a confirmed 2:1 ratio with benzathine, and should be considered in the context of the assay coefficient of variation (SD/mean) for the measured analyte (penicillin G) being 2.5% and potential systematic errors associated with aliquot measurements.

^b^ Reference range of the API is 123‐124° (The Merck Index). Melting point was determined on the BPG powder for injection.

^c^ D50 is the 50% point of the particle size distribution and represents the median diameter of the particles in the suspension. D10 and D90 are the 10% and 90% points of the distribution and are used to represent the range of the particle size distribution.

^d^ Samples 26 and 27 were the same batch sourced from two different countries

^e^ Sample 35 was analyzed three months after the stated expiry date

#### 2.2.2 Bicillin L‐A^®^ Suspension

Three aliquots (50 µL; 22.1 mg Bicillin L‐A^®^; measured by positive displacement pipette) were each dissolved with sonication in 2.5 mL DMF. A 500 µL aliquot of this solution was combined with 1.5 mL DMF and made up to a volume of 20 mL with water. The nominal concentration of BPG test solutions for analysis was 0.22 mg/mL in 10% v/v DMF in water. This method was used to confirm the HPLC assay was stability‐indicating and to process the aliquots for thermal stability assessment of BPG suspension (Bicillin L‐A^®^). Aliquots for the thermal stability study were stored in microcentrifuge tubes at 4°C (laboratory refrigerator), 25°C (room temperature) and 35°C (incubator), and analyzed at 0, 1, 3, 7, 10, 14, 20 and 26 weeks (see Figure S4).

### 2.3 Pharmaceutical quality tests

#### 2.3.1 Particle size analysis

A selection of 20 samples was subject to particle size analysis (Malvern Mastersizer 2000; Malvern Instruments Ltd, Malvern, UK). BPG vials were reconstituted in accordance with the manufacturer's recommendation and an aliquot (approximately 1 mL) was added to the Mastersizer dispersion unit for analysis.

#### 2.3.2 Melting point

The melting point of a selection of 20 samples of BPG powder for injection was performed by two independent operators (Branstead/Electrothermal Digital Melting Point Apparatus, Model 9100; Cole Parmer Ltd, Staffordshire, United Kingdom).

#### 2.3.3 Light microscopy

Qualitative inspection of BPG crystals was performed on a selection of 20 samples. A small aliquot of the powder for injection was gently suspended in water and placed on a standard microscope slide with glass coverslip for observation at 400 × magnification (Leica DM/LS microscope with Leica DC100 camera; Leica Microsystems Pty Ltd, Macquarie Park, NSW, Australia).

#### 2.3.4 Water content

The water content of a selection of 11 samples was conducted by a chemical analysis company, using the Karl‐Fischer coulometric titration method (Epichem Pty Ltd, Bentley, WA, Australia).

#### 2.3.5 Heavy metal analysis

Heavy metal analysis (As, Cd, Co, Cu, Hg, Li, Ni, Pb, Sb, V) of a selection of 11 samples was conducted by a chemical analysis company, using inductively coupled plasma optical emission spectrometry (ICP‐OES; Epichem Pty Ltd, Bentley, WA, Australia).

### 2.4 Statistical analysis

Data analysis and representation were performed with SigmaPlot v13 (Systat Software, San Jose, CA). Data are mean ± standard deviation (SD) unless otherwise indicated.

### 2.5 Ethics approval

Human Ethics approval was not required for this study.

## 3 RESULTS

### 3.1 Characteristics and origins

In total, 35 batches of BPG powder for injection were obtained from 16 countries (Table 1). These batches came from eight different pharmaceutical manufacturers. Two countries provided the same batch from the same pharmaceutical manufacturer, indicative of common distributor or small orders. All samples had a 3 year expiration from manufacture, except one—which had a 12 month expiration. Four Chinese pharmaceutical manufacturers were responsible for 29 of the batches (18, 6, 5, and 1, respectively), with the remainder coming from India, Pakistan, and United Arab Emirates. Eighteen vials contained 2.4 million international units (MIU) and seventeen contained 1.2 MIU.

Whilst repeated efforts were made to obtain BPG from all WHO regions, only four of the six WHO regions were represented in the present study: Africa (15 batches), Western Pacific (15), Eastern Mediterranean (3), and South‐East Asian (2), with no vials sourced from Europe or the Americas. Importantly, we obtained samples from regions where events had been reported. Batch 31 was obtained after reported adverse events (deaths) following administration of the same batch. Batch 11 was obtained from a region with recent reported adverse events, however, it is not known if there was an association with the tested batch. Batches 2 and 3 originated from the same country where there were reports of increased needle blockage. In order to maintain source anonymity, further details are not disclosed.

### 3.2 Content of commercial powdered BPG products

The nominal content of the 35 batches of BPG ranged from 91.5 ± 1.1% to 108.1 ± 7.4% (Table 1). There was no significant difference in content between the three principal pharmaceutical manufacturers’ products, at 102.3 ± 3.5% (n = 18), 101.7 ± 3.1% (n = 6), and 100.8 ± 5.2% (n = 5), respectively.

All samples were within United States Pharmacopeia (USP) specifications for BPG injection (90%‐115%) and the typical ranges (90%‐110%) for antimicrobial powders for injection in the British Pharmacopoeia (BP) and USP (Table 1). No degradation products were detected in the HPLC chromatograms for any tested vials.

### 3.3 Pharmaceutical quality tests

#### 3.3.1 Particle size analysis

The 20 batches of BPG subject to particle size analysis were sourced from 11 different countries and originated from six different pharmaceutical manufacturers (Table 1). Two manufacturers were responsible for 14 of the 20 tested batches, which had a mean (± SD) specific surface area of 1.03 ± 0.08 m^2^/g (n = 4) and 0.62 ± 0.29 m^2^/g (n = 10), respectively, (*P* = .02; t‐test). The mean (± SD) D_50_ from the two manufacturers was 12.5 ± 3.7 µm and 24.4 ± 8.6 µm, respectively, (*P* = .02; t‐test).

Two batches demonstrated a small proportion (<5%) of the particles being in the range of 400‐2000 µm (Figure S5 shows one sample). After 2‐3 minutes of vortex mixing, a secondary peak at ~ 1000 µm in the particle size distribution disappeared, implying the presence of aggregated particles, possibly due to caking and incomplete suspension of the powder upon addition of diluent.

Further investigation of the impact of sonication on particle size was conducted with two vials from a different batch of BPG powder for injection, whereby the powder was suspended according to the manufacturer's guidelines and subject to particle size analysis, then vortexed for one minute and sonicated for one minute, with particle size analysis at each stage. A modest reduction in D_50_ was observed after vortex mixing, from 7.5 µm to 7.2 µm in one vial and no change in the second vial. There was no improvement in the span of the particle size distribution, as determined from Span = (D_90_‐D_10_)/D_50_. However, sonication reduced D_50_ from 7.5 µm to 5.6 µm in one vial and 8.2 µm to 6.3 µm in the second vial. The span of the particle size distribution was reduced by 15% (2.05 to 1.75) and 12% (2.15 to 1.90) in the two vials (see Figure S6).

#### 3.3.2 Melting point

The mean ± SD (range) of initial melting points of the 35 batches of BPG powder for injection was 127 ± 2.5°C (123‐133°C). There was no significant difference in melting point between the three principal pharmaceutical manufacturers’ products, at 127.3 ± 2.4°C (n = 18), 128 ± 3.6°C (n = 6), and 125.2 ± 1.6°C (n = 5), respectively. Although it was outside the scope of the present study to extract the API from the BPG injections, these data indicate consistency of the products and are comparable to the API melting point (123‐124°C; Table 1), notwithstanding the likely influence of excipients.

#### 3.3.3 Light microscopy

Qualitative observations linked to the particle size analysis showed visible differences in the size and distribution of crystals (Figure S7). Our observations suggest that oblong shaped, plate‐like crystals were dominant, while evidence of needle‐like crystals was inconclusive.

#### 3.3.4 Water content

The water content of all 11 tested batches was within the BP and USP specification for BPG (API) of 5%‐8% (Table S1).

#### 3.3.5 Heavy metal analysis

The heavy metal analysis showed that all 11 batches were within the permitted concentrations specified by the Food and Drug Administration (Table S1).

## 4 DISCUSSION

This is the first study to assess the quality of commercially available powdered BPG sourced from multiple regions. All samples met the USP standards (BPG content 90%‐115%), without observed degradation products, including the one tested 3 months past expiration (#35; 95.8%). We tested samples from four of the six WHO regions, including Africa and the Western Pacific, regions where RHD continues to be major health concern.^18^ As our investigation focussed on the quality of BPG for community‐based end users in a range of international clinical settings, no information was available on the conditions for storage and transportation of vials prior to acquisition for the present study. These data should provide reassurance for clinicians across the globe who are responsible for managing the 19.9 million cases of syphilis, and 34 million people living with RHD.^18^, ^19^

Adverse events following BPG are rare. Given such reactions generally occur in patients with severe valvular pathology, the disease process itself is much more likely to be responsible.^8^ If BPG had an unidentified immunogenic contaminant/s we would expect significant clusters of events, which have not been reported. The BPG samples were assessed for the presence of penilloic acid, a recognized, immunogenic breakdown product of penicillin G. One batch of BPG had been linked to reported severe adverse events, but had satisfactory quality. Thus, the issue of adverse reactions may not be addressed by stricter controls on manufacturing process, as there do not appear to be significant quantities of low potency/quality BPG in clinical settings. Nevertheless, half of the tested batches (n = 18) were 2.4 MIU vials, which are typically subject to dose‐splitting and could become contaminated at the point of care.^20^

Water content and heavy metal testing were considered to be surrogate indicators of the manufacturing process (GMP) and storage of BPG injections. All 11 batches passed the water content specifications (a high water content could have occurred in the setting of poor storage of vials or inadequate compounding/packaging) environment. All tested batches also passed heavy metal analysis, although variation between batches was noted. High levels of heavy metals may reflect contaminated or poor quality manufacturing/packaging environments and whilst there is a known association with heavy metal exposure and certain clinical syndromes, the highest detected concentrations were typically at least one order of magnitude lower than the permissible FDA requirements for injectable medications.^21^

The low water solubility of BPG results in manufacturers adding emulsifying agents to improve mixing and suspension after reconstitution with an aqueous diluent. Lecithin, which is present in Bicillin L‐A^®^ (and some batches of BPG powder for injection), has been linked previously to adverse events, although testing to prove such an allergy is often not available in the low resource setting.^22^ We are aware of an individual likely allergic to an excipient (carmellose sodium, an emulsifier), resulting in rash with several brands yet able to tolerate other brands of BPG, without incident.^23^

A potentially clinically relevant finding in the present study relates to particle size and physical characteristics of BPG crystals. Although a previous study has demonstrated that crystal shape could contribute to needle blockages, all 20 samples we tested had oblong‐shaped crystals. We did however, demonstrate a substantial difference in particle size (Table 1 and Figure S7). The significant difference in particle size distribution (D_50_ 12.5 µm *vs* 24.4 µm) between two of the manufacturers and the observed clumping in 10% of samples tested (aggregation or incomplete suspension of powder, which was improved by sonication) indicated some heterogeneity in the BPG crystals, despite these batches meeting a range of standard pharmacopeial requirements. Clumping of particles may explain the issue of needle blockage, which has led to the use of excessive diluent volumes (up to 10 mL) for fear of wastage,^8^ as well as concerns about inferior quality of the BPG powder for injection.^10^, ^16^ As the elimination of clumping or caking of the powder and a tighter particle size distribution may improve delivery (syringeability), sonication of the reconstituted BPG immediately prior to injection could be an achievable solution for many clinical settings.

Whilst our study is the largest documented analysis of BPG powder for injection to date, products from two of the six WHO regions (Europe and the Americas) have not been examined. In addition, it was not feasible to detect all potential contaminants or degradation products, in our HPLC assay. Hence, we can only conclude that a range of commercially available BPG injections from the four WHO regions adhere to pharmacopeial standards. Furthermore, we cannot conclude that all commercially available BPG injections adhere to pharmacopeial standards, nor can we provide direct evidence that crystal size affects the syringeability or pharmacokinetic properties of BPG.

## 5 CONCLUSION

All batches of BPG we tested appeared to be of adequate potency and pharmaceutical quality, without detectable breakdown/contamination. This should give clinicians, public health officials, policymakers, and distributors confidence in the current supplies of BPG. However, we recommend improvements in BPG pharmacopeial monographs (API/products), including specifications on physical characteristics (eg, particle size, surface area, and crystal morphology). We also recommend further studies on the relationship between BPG crystal characteristics and pharmacokinetic properties, as well as patient and administration factors to reduce the risk of adverse events and improve tolerability of the BPG injection.

## DISCLOSURES

The authors have no conflicts of interest to declare.

## AUTHOR CONTRIBUTIONS

DSP, JC, KB, KG, LM, MS, RH, and RW contributed to study concept, protocol, and manuscript preparation. GL, GS, JM, MPS, and VC contributed to data collection, analysis, and manuscript preparation.

## Supporting information

Supplementary Material

## Data Availability

The data that support the findings of this study are available on request from the corresponding author. The data are not publicly available due to privacy or ethical restrictions.
