# Supplementary material for: Quality of benzathine penicillin G: A multinational cross‐sectional study
Source: Pharmacol Res Perspect. 2020 Oct 22;8(6):e00668. doi: 10.1002/prp2.668 (PMC7580708; doi:10.1002/prp2.668)
Supplement: Supplementary file 1 — Supplementary Material [file PRP2-8-e00668-s001.docx]

**Supplementary Information**

Quality of benzathine penicillin G: a multinational cross sectional study

Robert M. Hand,^1^ S.M.D.K. Ganga Senarathna,^2^ Madhu Page-Sharp, ^2^ Katherine Gray,^1^ Dianne Sika-Paotonu,^1,3,4,5^ Meru Sheel,^1^ Victor T.G. Chuang,^6^ Jorge Martinez,^2^ Giuseppe Luna,^2^ Laurens Manning,^1,7^ Rosemary Wyber,^1,8^ Jonathan R. Carapetis,^1,9^ Kevin T. Batty^2#^

^1^Wesfarmers Centre of Vaccines and Infectious Diseases, Telethon Kids Institute, University of Western Australia, Perth, Western Australia

^2^School of Pharmacy and Biomedical Sciences, Curtin University, Bentley, Western Australia

^3^Dean’s Department and Department of Pathology & Molecular Medicine, Wellington School of Medicine and Health Sciences, University of Otago, New Zealand

^4^Faculty of Health, Victoria University of Wellington, New Zealand

^5^Maurice Wilkins Centre for Molecular Biodiscovery, University of Auckland, New Zealand

^6^School of Pharmacy, Monash University Malaysia, Selangor, Malaysia

^7^Faculty of Health and Medical Sciences, University of Western Australia, Perth, Western Australia

^8^The George Institute for Global Health, Sydney, New South Wales

^9^Department of Infectious Diseases, Perth Children’s Hospital, Perth, Western Australia

**Benzathine penicillin G information**

Benzathine penicillin G (BPG) is a crystalline combination of penicillin G and benzathine (2:1 molar ratio). BPG is a white, odourless, crystalline powder; it is very slightly soluble in water, freely soluble in dimethylformamide and slightly soluble in ethanol (96% v/v).

BPG is available from a range of pharmaceutical manufacturers as a powder for injection (suspended with a diluent prior to intramuscular injection) and as an aqueous suspension (Bicillin L-A^®^) which contains 0.5% w/v lecithin, 0.6% w/v carboxymethylcellulose and 0.6% w/v povidone (Pfizer Australia Pty Ltd, West Ryde NSW, Australia; Prescribing Information 2017).

Manufacturers of BPG powder for injection recommend reconstitution with variable volumes (6-10 mL) of Water for Injection. Sterile water may be substituted with lignocaine injection to reduce the pain of intramuscular injection, without apparent alteration of pharmacokinetic profile^^[[1]](#footnote-1)^^.

Bicillin L-A^®^ is widely used in countries with reliable cold-chain storage and distribution (Australian recommendations require refrigeration at 2-8°C until day of use), although Canadian guidelines indicate the product can be kept at room temperature for 7 days (<30°C)^^[[2]](#footnote-2)^^. However, in our experience, clinical staff discard the medication if not administered same-day, due to perceived instability, and we are also aware of instances where power failures have resulted in cold chain failure and stock being discarded.

**Benzathine penicillin G assay**

***Materials and reagents***

Benzathine penicillin G tetrahydrate (C_32_H_36_N_4_O_8_S_2_.C_16_H_20_N_2_.4H_2_0; MW 981; BPG), potassium phosphate monobasic (KH_2_PO_4_; MW 136) and N,N’-Dibenzylethylenediamine (C_16_H_20_N_2_; MW 240; Benzathine) were purchased from Sigma Aldrich, St Louis, MO, USA. Penicillin G sodium CRS (C_16_H_17_N_2_NaO_4_S; MW 356) was provided by the European Pharmacopoeia Reference Standards, EDQM-Council of Europe, Strasbourg, France. Penilloic acid of penicillin G (C_15_H_20_N_2_O_3_S; MW ‎308) was purchased from LGC GmbH, Luckenwalde, Germany. 1-Heptane sulfonic acid sodium (C_7_H_15_SO_3_Na; MW 202) was obtained from Fisher Scientific UK, Loughborough, Leicestershire, UK. N,N-Dimethylformamide (C_3_H_7_NO; MW 73; DMF) was obtained from Ajax Finechem, Taren Point, NSW, Australia. HPLC grade acetonitrile was purchased from Scharlab S.L., Sentmenat, Barcelona, Spain. Benzathine penicillin G injection (Bicillin L-A^®^; Pfizer Australia Pty Ltd, West Ryde, New South Wales, Australia) was obtained from a commercial pharmacy source in Australia. All other laboratory chemicals were of analytical grade.

***High performance liquid chromatography assay***

The analysis was performed using an Agilent 1200 series high performance liquid chromatography (HPLC) system comprising a binary gradient pump with a degasser, autosampler, a thermostated column oven (34°C) and a dual wavelength UV detector (Agilent Technology, Waldbronn, Germany). Chemstation B.03.01.SR1 software (Agilent) was used to process and analyse the data.

A reversed-phase, ion-pairing HPLC method^^[[3]](#footnote-3)^^ with minor modifications was adapted for the present study. Separation was performed using a Phenomenex^®^ Gemini C_6_ Phenyl (5 µm, 4.6 × 150 mm) HPLC column. The mobile phase comprised KH_2_PO_4_ (32 mM) and ion paring agent, 1-heptane sulfonic acid sodium (31 mM) in de-ionised water (71%) and acetonitrile (29%), adjusted to pH 3.17 using dilute phosphoric acid. The mobile phase was pumped at 1.2 mL/min and the analytes were detected at 214 nm. The injection volume was 40 µL. Retention times for penicillin G and benzathine were 5.9 and 7.4 minutes, respectively (Supplementary Figure S1). As benzathine penicillin G (BPG) dissociates in solution, the peak area of penicillin G was used for quantification of BPG. The presence of the degradation product, penilloic acid, was determined using the same HPLC method as for BPG.

Stock solutions were prepared in DMF and standard series were diluted in water, maintaining 10% v/v DMF in the final solution. Calibration curves for penicillin G and benzathine were constructed at concentrations of 0.0125 to 0.4 mg/mL.

Chromatograms for penicillin G, benzathine, benzathine penicillin G and penilloic acid of penicillin G are shown in Figure S1 (panels A to D respectively) and demonstrate acceptable selectivity of the HPLC assay. The chromatogram for forced-degradation of BPG solution (Bicillin LA^®^; 1:2000 in 10% DMF) stored at 35°C for 2.5 days is shown in Figure S2. The calibration curve for benzathine penicillin G, which can be quantified from the penicillin G and/or benzathine peaks, is provided in Figure S3. The limits of detection and quantification were 1 and 2 µg/mL respectively for penicillin G, and 2 and 5 µg/mL for benzathine. Inter- and intra-run coefficients of variation for penicillin G were 1% and 2.1% at 0.0125 mg/mL, 1.6% and 2.4% at 0.05 mg/mL and 0.5% and 2.5% at 0.2 mg/mL, respectively. Inter- and intra-run coefficients of variation for benzathine were 8.1% and 10.4% at 0.0125 mg/mL, 2.9% and 6.7% at 0.05 mg/mL and 2.1% and 3.3% at 0.2 mg/mL, respectively. The calibration curve for penilloic acid was linear from 0.8 to 200 µg/mL (r^2^ = 0.999) and the limit of detection was 0.4 µg/mL.

**Figure S1.** Chromatograms for penicillin G (Panel A; t_R_ = 6.1 min; 0.48 µmol/mL; 0.16 mg/mL), benzathine (Panel B; t_R_ = 7.4 min; 0.24 µmol/mL; 0.058 mg/mL), benzathine penicillin (Panel C; 0.24 µmol/mL; 0.22 mg/mL, showing peaks for penicillin G and benzathine) and penilloic acid of penicillin G (Panel D; t_R_ = 3.3 min; 0.04 µmol/mL; 12.5 µg/mL).

| **A** | 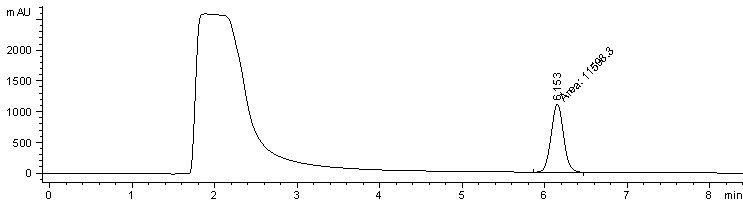 |
| --- | --- |

| **B** | 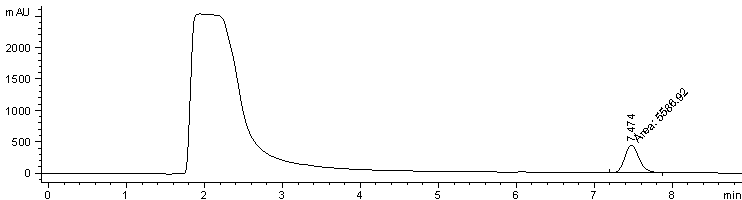 |
| --- | --- |

| **C** | 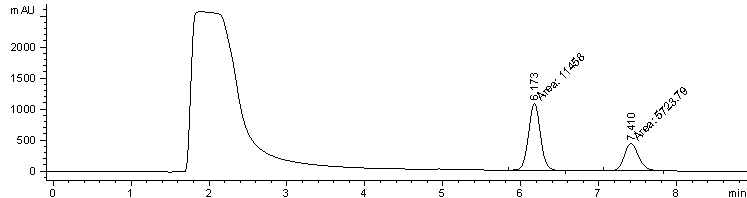 |
| --- | --- |

| **D** | **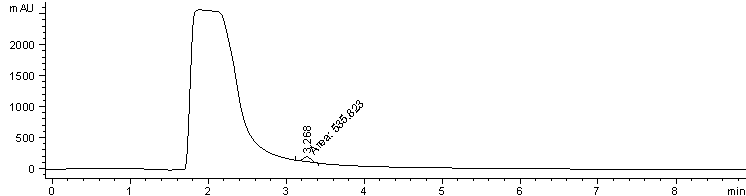** |
| --- | --- |

**Figure S2.** Chromatogram of forced-degradation of BPG.

Bicillin L-A^®^ 0.22 mg/mL in 10% v/v dimethylformamide in water was stored at 35°C for 2.5 days. The stability-indicating assay shows penicillin G (t_R_ = 6.1 min), benzathine (t_R_ = 7.4 min), penilloic acid (t_R_ = 3.3 min) and an unidentified compound (t_R_ = 2.8 min). The unidentified compound was not further investigated as it was not detected in any chromatograms associated with the purity assessment or Bicillin L-A^®^ stability study (penilloic acid also was not detected in the stability study). *Note*: The rapid degradation of a dilute solution of BPG (in 10% v/v DMF in water) is in contrast to the prolonged stability of (undiluted) Bicillin L-A^®^ at room temperature and 35°C - see Figure S4.


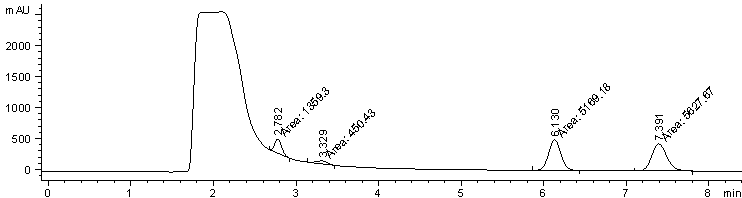


**Figure S3.** Calibration curve for benzathine penicillin (0.0125 to 0.4 mg/mL), quantified using penicillin G (●; r^2^>0.999) and/or benzathine (🞅; r^2^>0.999) peaks.

**Figure S4**. Thermal stability of commercial benzathine penicillin G suspension injection (Bicillin LA®).

Aliquots (50 µL) were stored in microcentrifuge tubes at 4°C (●; laboratory refrigerator), 25°C (○; room temperature) and 35°C (△; incubator), and analysed at 0, 1, 3, 7, 10, 14, 20 and 26 weeks. Data are mean ± SD. First-order regression was performed on the drug content-time data at each temperature (4°C ― and 25°C ‐‐‐ are shown). As there was no apparent degradation for the duration of the study, and the 95% CI at each time point spanned the assayed content at zero time for all three temperatures, it was concluded that Bicillin L-A^®^ was stable for at least six months at temperatures ranging from 4-35°C. In order to reduce unnecessary disposal of Bicillin L-A^®^ injection that has been stored at room temperature for up to one week, we concur with Canadian guidelines indicating the product can be kept at room temperature (<30°C) for 7 days (https://www.pfizer.ca/sites/default/files/201902/Bicillin-LA_PM_E_222142_23Jan2019.pdf).

**Figure S5**. The effect of vortex mixing on the particle size distribution of BPG powder suspended in water, according to the manufacturer’s recommendations (Panel A). In this sample, the secondary peak indicates a small proportion (<5%) of the particles were in the range of 400-2,000 µm. The suspension was subject to 2-3 minutes of vortex mixing, which eliminated the secondary peak at approximately 1,000 µm in the particle size distribution (Panel B).

**Panel A: Suspension**

**Panel B: Vortex mixing**

**Figure S6**. The effect of sonication on the particle size distribution of BPG powder suspended in water, according to the manufacturer’s recommendations (Panel A). A modest reduction in the D50 was observed after vortex mixing (not shown) and there was no improvement in the span of the particle size distribution, as determined from Span = (D90-D10)/D50. Following sonication for one minute, D50 was reduced from 7.5 µm to 5.6 µm and the span of the particle size distribution was reduced by 15% (2.05 to 1.75; Panel B).

**Panel A: Suspension**

**Panel B: Sonication**

**Figure S7**. Microscopy (400× magnification) of BPG powder suspended in water. Panels A and B show the crystals from two different batches with average particle size at 32 and 33 µm. Panels C and D show the smaller average particle size of 10 and 11 µm from another two batches.

| **Panel A:** D50 = 32 µm; D10-90 = 8-89 µm  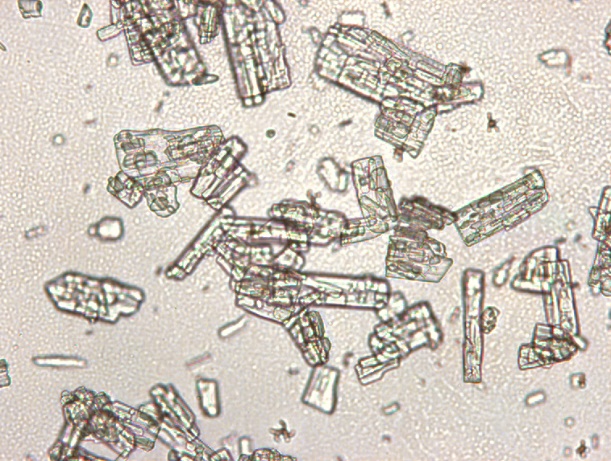 | **Panel B:** D50 = 33 µm; D10-90 = 5-86 µm  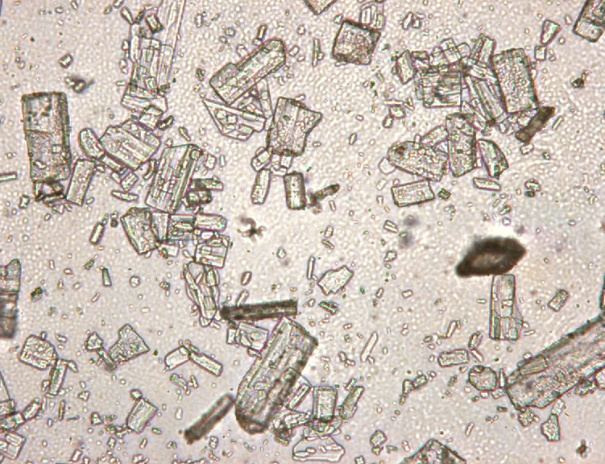 |
| --- | --- |
| **Panel C:** D50 = 10 µm; D10-90 = 3-34 µm  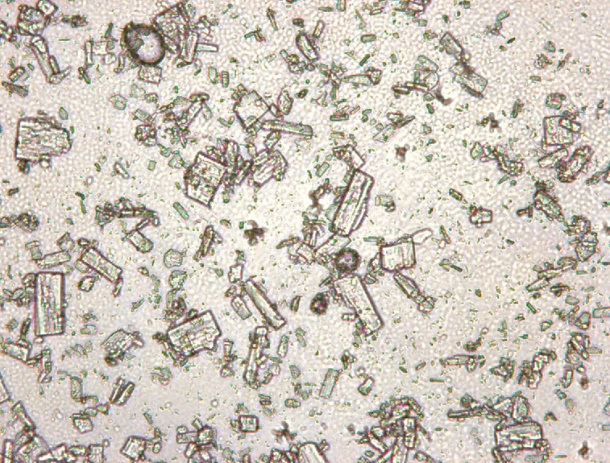 | **Panel D:** D50 = 11 µm; D10-90 = 3-36 µm  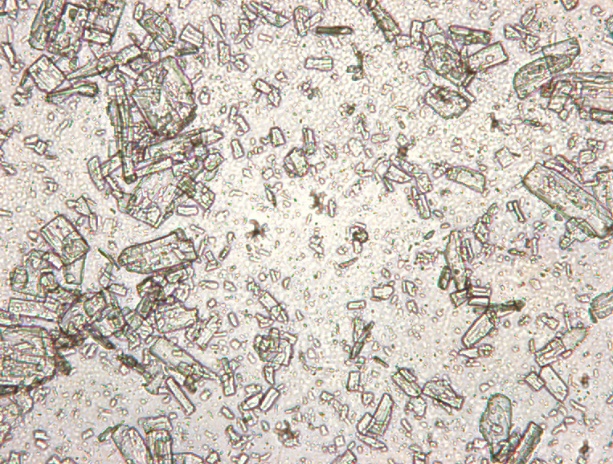 |

**Table S1**. Water content and heavy metal analysis of BPG injection powder sourced from international clinical settings.

| **Code*** |  | **Heavy metal (PPPM)^‡^** | | | | | | | | | |
| --- | --- | --- | --- | --- | --- | --- | --- | --- | --- | --- | --- |
|  | **H_2_O^†^** | **As** | **Cd** | **Co** | **Cu** | **Hg** | **Li** | **Ni** | **Pb** | **Sb** | **V** |
|  | **(5-8%)** | **<1.5** | **<0.2** | **<0.5** | **<30** | **<0.3** | **<25** | **<2** | **<0.5** | **<9** | **<1** |
| 1 | 7 | 0.003 | <0.005 | <0.002 | 4.448 | 0.014 | <0.005 | <0.005 | 0.005 | <0.005 | <0.003 |
| 2 | 8 | 0.044 | <0.005 | 0.006 | 0.078 | <0.005 | 0.098 | 0.022 | 0.026 | <0.005 | 0.008 |
| 3 | 7 | 0.084 | 0.084 | 0.007 | 1.469 | <0.005 | 0.192 | 0.02 | 0.022 | <0.005 | 0.017 |
| 11 | 7 | 0.049 | <0.005 | 0.002 | 0.071 | <0.005 | <0.005 | 0.055 | 0.041 | <0.005 | 0.007 |
| 14 | 6 | 0.234 | 0.031 | 0.032 | 3.176 | <0.005 | 0.016 | 0.062 | 0.055 | 0.048 | 0.063 |
| 20 | 7 | <0.003 | <0.005 | <0.002 | 0.861 | <0.005 | <0.005 | 0.033 | 0.006 | <0.005 | <0.003 |
| 21 | 7 | 0.011 | <0.005 | <0.002 | 0.017 | 0.112 | 0.031 | 0.011 | 0.016 | <0.005 | <0.003 |
| 22 | 7 | 0.322 | 0.053 | 0.053 | 0.142 | <0.005 | 0.039 | 0.158 | 0.105 | 0.075 | 0.088 |
| 23 | 8 | 0.006 | <0.005 | <0.002 | 7.161 | 0.052 | 0.013 | 0.008 | 0.005 | <0.005 | <0.003 |
| 24 | 8 | 0.024 | <0.005 | <0.002 | 0.058 | 0.167 | <0.005 | 0.04 | 0.008 | <0.005 | <0.003 |
| 31 | 6 | 0.005 | 0.03 | <0.001 | 0.315 | <0.001 | 0.009 | 0.019 | 0.013 | 0.017 | <0.001 |

* Codes as indicated in Table 1. Samples 1-3 and 22-24 from the same manufacturer (all different batches). All other samples from different manufacturers.

^†^ Water content: Reference range 5-8% (British Pharmacopoeia).

^‡^ PPPM: permitted parts per million (FDA injectables); As - Arsenic, Cd - Cadmium, Co - Cobalt, Cu - Copper, Hg – Mercury, Li – lithium, Ni – Nickel, Pb - Lead, Sb – Antimony, V - Vanadium

1. Amir J, Ginat S, Cohen YH, Marcus TE, Keller N, Varsano I 1998. Lidocaine as a diluent for administration of benzathine penicillin G. Pediatr Infect Dis J 17(10):890-893. [↑](#footnote-ref-1)
2. https://www.pfizer.ca/sites/default/files/201902/Bicillin-LA_PM_E_222142_23Jan2019.pdf [↑](#footnote-ref-2)
3. Irwin WJ, Hempenstall JM, Li Wan Po A 1984. Controlled-release penicillin complexes. High-performance liquid chromatography and assay. J Chromatogr 287(1):85-96. [↑](#footnote-ref-3)
